# Lipopolysaccharide induces retention of E-cadherin in the endoplasmic reticulum and promotes hybrid epithelial-to-mesenchymal transition of human embryonic stem cells-derived expandable lung epithelial cells

**DOI:** 10.1007/s00011-025-02041-4

**Published:** 2025-05-24

**Authors:** Türkan Portakal, Vítězslav Havlíček, Jarmila Herůdková, Vendula Pelková, Tereza Gruntová, Rıza Can Çakmakci, Hana Kotasová, Aleš Hampl, Petr Vaňhara

**Affiliations:** 1 Department of Histology and Embryology, Faculty of Medicine, Masaryk University, Kamenice 753/5, 625 00 Brno, Czech Republic; 2 International Clinical Research Center, St. Anne’s University Hospital, Pekařská 664/53, 602 00 Brno, Czech Republic; 3 University Hospital Brno, Jihlavská 340/20, 625 00 Brno, Czech Republic

**Keywords:** Lipopolysaccharide, Unfolded protein response, Epithelial-to-mesenchymal transition, Expandable lung epithelium

## Abstract

**Background:**

Lipopolysaccharide (LPS)-induced inflammation of lung tissues triggers irreversible alterations in the lung parenchyma, leading to fibrosis and pulmonary dysfunction. While the molecular and cellular responses of immune and connective tissue cells in the lungs are well characterized, the specific epithelial response remains unclear due to the lack of representative cell models. Recently, we introduced human embryonic stem cell-derived expandable lung epithelial (ELEP) cells as a novel model for studying lung injury and regeneration.

**Methods:**

ELEPs were derived from the CCTL 14 human embryonic stem cell line through activin A-mediated endoderm specification, followed by further induction toward pulmonary epithelium using FGF2 and EGF. ELEPs exhibit a high proliferation rate and express key structural and molecular markers of alveolar progenitors, such as NKX2-1. The effects of Escherichia coli LPS serotype O55:B5 on the phenotype and molecular signaling of ELEPs were analyzed using viability and migration assays, mRNA and protein levels were determined by qRT-PCR, western blotting, and immunofluorescent microscopy.

**Results:**

We demonstrated that purified LPS induces features of a hybrid epithelial-to-mesenchymal transition in pluripotent stem cell-derived ELEPs, triggers the unfolded protein response, and upregulates intracellular β-catenin level through retention of E-cadherin within the endoplasmic reticulum.

**Conclusions:**

Human embryonic stem cell-derived ELEPs provide a biologically relevant, non-cancerous lung cell model to investigate molecular responses to inflammatory stimuli and address epithelial plasticity. This approach offers novel insights into the fine molecular processes underlying lung injury and repair.

**Supplementary Information:**

The online version contains supplementary material available at 10.1007/s00011-025-02041-4.

## Introduction

Exposure of lungs to pathogens, environmental pollutants, or toxins evokes a complex inflammatory response in lung tissue that involves recruitment and activation of immune cells, release of pro-inflammatory cytokines, and alterations in vascular permeability. Massive or long-term inflammation then leads to structural alterations in the alveolar parenchyma, decreased gas exchange, and ultimately to systemic effects such as hypoxemia and exacerbation of other comorbidities. The inflammation-driven structural changes in the lungs involve the accumulation of extracellular matrix, disruption of the alveolar-capillary barrier, and increased cellular plasticity within the alveolar compartment [1, 2].

Lipopolysaccharide (LPS, endotoxin) is a large amphipathic molecule located in the outer membrane of Gram-negative bacteria. It consists of three main domains that contribute to the stability of the bacterial membrane and, through structural variability, enable immune evasion while simultaneously triggering a potent inflammatory response in infected tissues through recognition of pathogen-associated molecular patterns (PAMPs), preferentially by macrophages and dendritic cells [3].

LPS binds to the CD14 molecule on immune cells, forming a complex with toll-like receptor 4 (TLR4) and the co-receptor, myeloid differentiation factor 2 (MD-2). The canonical LPS signaling pathway then involves the recruitment of intracellular adaptor proteins, e.g. MyD88 and TRIF, and canonically activates the NF-κB transcription factor [4, 5]. In the lungs, this signaling cascade triggers the release of inflammatory mediators, including cytokines, chemokines, and antimicrobial peptides, which contribute to the inflammatory response and the recruitment of immune cells to the site of infection [6]. Additionally, endothelial cells and macrophages are activated to produce mediators that increase vascular permeability, promoting neutrophil infiltration. Moreover, the type II alveolar pneumocytes (granular pneumocytes) are also activated, leading to surfactant production and their trans-differentiation into type I pneumocytes (membranous pneumocytes) to restore the alveolar-capillary barrier [7] and alveolar repair [8]. Altogether, exposure of lungs to LPS evokes a complex molecular response leading to structural and functional alterations.

Epithelial-mesenchymal transition (EMT) is a key process in tissue regeneration, including lungs. EMT involves the acquisition of migratory and secretory capabilities by epithelial cells, and molecular switch of cell adhesion molecules, e.g. E- and N-cadherins. The imbalance between E-cadherin and N-cadherin enhances cell migration and proliferation in the alveolar compartment [9]. EMT-associated transcription factors, e.g. SNAI1 (Snail), promote EMT by downregulating epithelial adhesion molecules, driving the fibrotic response and influencing lung tissue regeneration [10]. However, EMT can contribute to excessive lung tissue remodeling, deposition of ECM, and development of pulmonary fibrosis [11]. Hybrid EMT was proposed recently to explain the phenotypic variability of metastasizing cancer cells displaying markers of both mesenchymal and epithelial phenotypes (for review see [12]. Hybrid EMT was also associated with tissue plasticity and dynamic transition between mesenchymal and epithelial states by mathematical modeling [13] and validated in various biological experimental designs of metastasizing cancer including breast cancer [14], skin squamous cell carcinoma [15] or lung cancer [16].

The endoplasmic reticulum (ER) is a principal organelle dedicated for synthesis and post-translational modification of membrane and secreted proteins. It also represents an important signaling hub integrating various extrinsic and intrinsic cues that can compromise proteosynthesis and induce ER-stress [17]. The unfolded protein response (UPR) is a collective term for integrated molecular signaling induced during ER stress. UPR either induces programmed cell death or restores ER homeostasis by attenuating protein translation, enhancing the degradation of misfolded proteins, and upregulating the expression of chaperones [18, 19]. Induction of prosurvival UPR was associated with EMT in various cell types, including lung cancer [20].

Although the cellular responses of immune and connective tissue cells to LPS are well-documented, the mechanisms underlying the epithelial response remain poorly understood, primarily due to the absence of effective cellular models. Most of the publications are fundamentally linked to lung pathologies, such as cancer, pulmonary fibrosis, or chronic obstructive pulmonary disease. To investigate the stress response and cell plasticity of healthy, non-cancerous lung cells, we have derived the expandable lung epithelial (ELEP) cells derived from human embryonic stem cells (hESCs). ELEPs express markers of lung progenitors, such as NKX2-1, produce lamellar bodies, and possess the capacity to differentiate into various lung epithelial cell phenotypes in vivo and in vitro [21]. The ELEPs can be easily propagated in culture and maintain the lung epithelial phenotype over tens of passages.

In this work, we describe for the first time that purified LPS from *E. coli* induces hallmarks of hybrid epithelial-to-mesenchymal transition of ELEP cells in vitro through the retention of E-cadherin in the endoplasmic reticulum and eliciting the UPR.

## Materials and methods

### Cell culture and treatments

The ELEPS were differentiated as described previously [21]. Briefly, MUNIe007-A (RRID:CVCL_C860) hESC line [46, XX, CCTL 14] was used for derivation of ELEPs (for a detailed characterization of the CCTL 14 line see Human Pluripotent Stem Cell Registry, https://hpscreg.eu, 15.2.2025). hESC CCTL 14 cells were used in consonance with legal permission issued by Ministry of Education, Youth and Sports of the Czech Republic (MSMT-19838/2022-7). hESCs were grown in a feeder-free culture in Dulbecco’s modified Eagle medium (DMEM)/F12 supplemented with 15% knockout serum replacement, 1 × MEM nonessential amino acids, 100 U/ml penicillin, 100 μg/ml streptomycin, 2 mM l-glutamine (all from Invitrogen/Thermo Fisher Scientific, USA), 10 ng/ml fibroblast growth factor 2 (FGF2) (PeproTech, Germany) and 0.1 mM β-mercaptoethanol Merck, Czech Republic). For passaging, hESCs were dissociated with trypsin into a single-cell suspension and plated on vitronectin-coated dishes (Nucleus Biologics, USA) at the density of 10 cells/cm^2^. For ELEP differentiation, hESCs were initially cultured in standard medium DMEM/F12 supplemented with 2 mM l-glutamine, 100 U/ml penicillin, 100 μg/ml streptomycin and 10% FBS. After 24 h, they were stimulated by 50 ng/ml activin A (PeproTech, Germany) for five days. Then the medium was changed to serum-free DMEM/F12 with 2 mM l-glutamine, 100 U/ml penicillin, 100 U/ml streptomycin, and 1 × insulin/ transferrin/selenium (ITS) (Gibco/Thermo Fisher Scientific, USA). Differentiating cells were then collected on day 8 and seeded on vitronectin-coated dishes at density 5 × 10^3^ cells/cm^2^ in ELEP propagation medium composed of DMEM/F12 supplemented with 2% FBS, 2 mM l-glutamine, 100 U/ml penicillin, 100 U/ml streptomycin, 1 × ITS, 5 μg/ml heparin (Merck, Czech Republic), 10 ng/ml FGF2 and 20 ng/ml epidermal growth factor (EGF) (PeproTech Germany). Lung cell identity of ELEPs was confirmed by determining the expression of NKX2-1 transcription factor. ELEPs were passaged every two days at 70% confluency. For experimenting, ELEPs at passage numbers 30–60 were used as described previously [21, 22]. Formation of 3D ELEP spheroids was induced by culturing suspension of 1000 cells in 25 μl hanging drops of ELEP propagation medium for 48 h. Lipopolysaccharide from *E. coli* serotype 055:B5 (L2637), sodium tauroursodeoxycholate (TUDCA, T0266), tunicamycin (T7765) and glycogen synthase kinase-3 inhibitor (CHIR99021, SML1046) were purchased from Sigma Aldrich/Merck (Czech Republic). Lung carcinoma cell line A549 (ATCC^®^ CCL-185™) was cultured in DMEM supplemented with 100 U/ml penicillin, 100 μg/ml streptomycin and 10% FBS and used as a positive control where necessary.

### Flow cytometry

ELEPs were dissociated with trypsin, fixed in 4% paraformaldehyde for 30 min, permeabilized for 10 min with 0.1% Triton X-100, and then incubated with primary anti-NKX2-1 polyclonal antibody (sc-13040, 1:100, Santa Cruz Biotechnology, USA, and ab76013, 1:100, Abcam, UK) overnight at 4 °C. For negative controls the cells were incubated with isotype control antibody (rabbit IgG, sc-2027, 1:200, Santa Cruz Biotechnology, USA). After three washes in 1 × PBS, the cells were incubated with secondary anti-rabbit antibody AlexaFluor 488 (A11008, 1:500, Invitrogen/Thermo Fisher Scientific, USA) for 1 h at RT. Cells were analyzed on a BD FACSCanto flow cytometer (Becton Dickinson, USA), equipped with laser 488. Dead cells, cell aggregates and debris were excluded from the analysis. The gates for NKX2-1 positivity were set based on isotype controls. Acquired FCS files were exported and analyzed using BD FACSDiva Software v6.1.2.

### MTT assay

ELEPs were seeded into a 96-well plate (Corning, Germany) at the 70% confluence and allowed to adhere overnight. Then, the medium was changed, and the ELEPs were exposed to varying concentrations of LPS. To determine cell viability through mitochondrial metabolic activity, the thiazolyl blue tetrazolium bromide (M2128, Sigma Aldrich/Merck, Czech Republic) solution in water (5 mg/ml) was added at one-tenth of culture volume and incubated at 37 °C for 4 h. Absorbance was measured upon cell lysis at 570 nm. The plots represent normalized, background subtracted mean absorbance ± standard deviation (SD).

### Migration assay

ELEPs were seeded into a 6-well plate (Corning, Germany) using 2-well culture inserts (ibidi, Germany) and allowed to adhere overnight. Alternatively, the gap was created manually using a sterile pipette tip. After removal of the inserts or manual scratching, the wells were rinsed with 1 × PBS to remove cell debris and floating cells and then incubated in fresh ELEP passaging medium for indicated time. Cell migration over the free area was captured at 24 and 48 h. timepoints using a light microscopy. The extent of gap closure was analyzed using ImageJ software [23].

### Immunofluorescence staining

Cells were washed in 1 × PBS, fixed in 4% paraformaldehyde for 15 min, washed in 1 × PBS, permeabilized in 0.1% Triton X-100 for 10 min, blocked with 1% bovine serum albumin (BSA) in PBS (pH 7.4) for 1 h. Then, cells were incubated overnight at 4 °C with the following primary antibodies diluted in 3% BSA in 1 × PBS: NKX2-1, sc-13040, 1:1000, Santa Cruz Biotechnology, USA. BiP, CS3177, 1:500, CHOP, CS2895, 1:500, Calnexin, CS2679S, 1:500, and E-cadherin, CS3195, 1:1000, all from Cell Signaling Technology, USA. N-cadherin, Ab98952, 1:1000, Abcam UK; TUSC3, 67382, 1:500, Proteintech, USA; The cells were then washed three times in 1 × PBS and incubated for 1 h with secondary antibody conjugated with AlexaFluor 568 (goat anti-mouse, A11036) or Alexa Fluor 488 (goat anti-rabbit, A11008), from Invitrogen /Thermo Fisher Scientific, USA), both diluted 1:2000 in 3% BSA/PBS followed by additional wash in 1 × PBS. DAPI diluted 1:2000 in 0.05% Tween-20/PBS was added to the final concentration 5 μg/ml. Samples were mounted using Roti-Mount FluorCare for microscopy. Images were acquired using Zeiss Axio Observer.Z1/7 equipped with Plan-Apochromat 63x/1.40 Oil DIC M27 objective.

### SDS-PAGE and western blotting

Cells were washed two times with ice-cold 1 × PBS and resuspended in lysis buffer containing 50 mM Tris–HCl (pH 7.5), 1% SDS, and 10% glycerol. Next, 15 μg protein extract, quantified using the Bradford-based BioRad protein Assay Kit (BioRad, USA), was mixed with 2 × Laemmli sample buffer (100 mM Tris pH 6.8, 4% SDS, 200 mM DTT, 20% glycerol, and 0.1% Bromophenol Blue), incubated for 5 min at 95 °C, and resolved using 10% sodium dodecylsulfate-polyacrylamide gel electrophoresis (SDS-PAGE). Resolved proteins were then electroblotted to a 0.45 μm polyvinylidene difluoride (PVDF) membrane (Millipore/Merck, USA), blocked in TBS buffer (20 mM Tris–HCl, pH 7.2, 140 mM NaCl, 0.1% Tween 20) containing 5% non-fat dry milk or BSA, and incubated with the following primary antibodies diluted at 4 °C overnight: NKX2-1, sc-13040, 1:1000, Santa Cruz Biotechnology, USA. TUSC3, 67382, 1:500, Proteintech, USA. BiP, CS3177, 1:500; CHOP, CS2895, 1:500; Calnexin, CS2679S, 1:500; E-cadherin, CS3195, 1:1000; N-cadherin, CS13116S and β-actin, CS4970, 1:1000, all from Cell Signaling Technology, USA. Blots were developed using horseradish peroxidase (HRP)-conjugated secondary IgG antibodies and Immobilon Western HRP Substrate (Millipore), according to the manufacturer’s protocols. Quantitative densitometry was performed using ImageJ software. Values above the bands represent the ratio of the integrated band density to the loading control.

### RNA isolation, cDNA synthesis, and qRT-PCR analysis

Total RNA was isolated from cells using RNA Blue reagent and quantified using NanoDrop (Thermo Scientific). cDNA synthesis was performed from 1 μg of RNA by the Applied Biosystems™ High-Capacity cDNA Reverse Transcription Kit according to the manufacturer’s instructions. SYBR green based quantitative real-time PCR (qRT-PCR) was performed on the Roche LightCycler^®^ 480 system. The qRT-PCR program included an initial activation step at 95 °C for 5 min, followed by 40 cycles of 95 °C for 10 s, annealing at 60 °C for 10 s, and extension at 72 °C for 10 s. Sequences of primers are provided in Supplementary Table 1. Gene expression levels were calculated based on the threshold cycle (Ct) values and normalized to glyceraldehyde-3-phosphate dehydrogenase (GAPDH) expression using the ΔΔCt method.

### Statistical analysis and morphometry

All experiments were performed in triplicate at a minimum. The number of experimental replicates is indicated in the figure legends. Where appropriate, a *t*-test was used to evaluate statistical significance, with a threshold set at *p* < 0.05. Segmentation of cell and nuclear images was performed manually using CellPose 2.0. [24]. Morphometric analysis was conducted using ImageJ [23]. All plots were generated in GraphPad software (Dotmatics, USA).

## Results

First, we differentiated the ELEPs from human embryonic stem cells (hESCs) according to our protocol published recently [21]. We confirmed ELEP cell identity by the expression of NK2 homeobox 1 (NKX2-1) gene specific for pulmonary epithelia, by immunofluorescent microscopy and flow cytometry. Upon differentiation from hESCs, the ELEPs acquired epithelial morphology, produced high levels of NKX2-1, and maintained the high proliferation rate as reported in [21] and confirmed in Figs. S1 and S2A.

To investigate the response of the ELEPs to bacterial LPS, we exposed them to increasing concentrations of purified LPS O55 from *E. coli*. Short-term exposure of up to 48 h of ELEPs to LPS did not compromise the cell survival and mitochondrial functions across a range of concentrations, as documented by the MTT assay (Fig. S2B). Interestingly, cell viability remained unaffected after several weeks of culture in the presence of 2 μg/ml LPS (Fig. S3B). However, the ELEP cell monolayer displayed a dispersed and rather loosened arrangement and increased total cell area upon treatment with LPS (Fig. S3A), but the cells have not developed any substantial changes in other gross cell morphology parameters, such as aspect ratio and circularity, as quantified by morphometric analysis (Fig. S3B). Analysis of nuclear morphology revealed an increased aspect ratio and reduced circularity, with the overall nuclear area remaining unchanged (Fig. S3C).

**Fig. 1:**
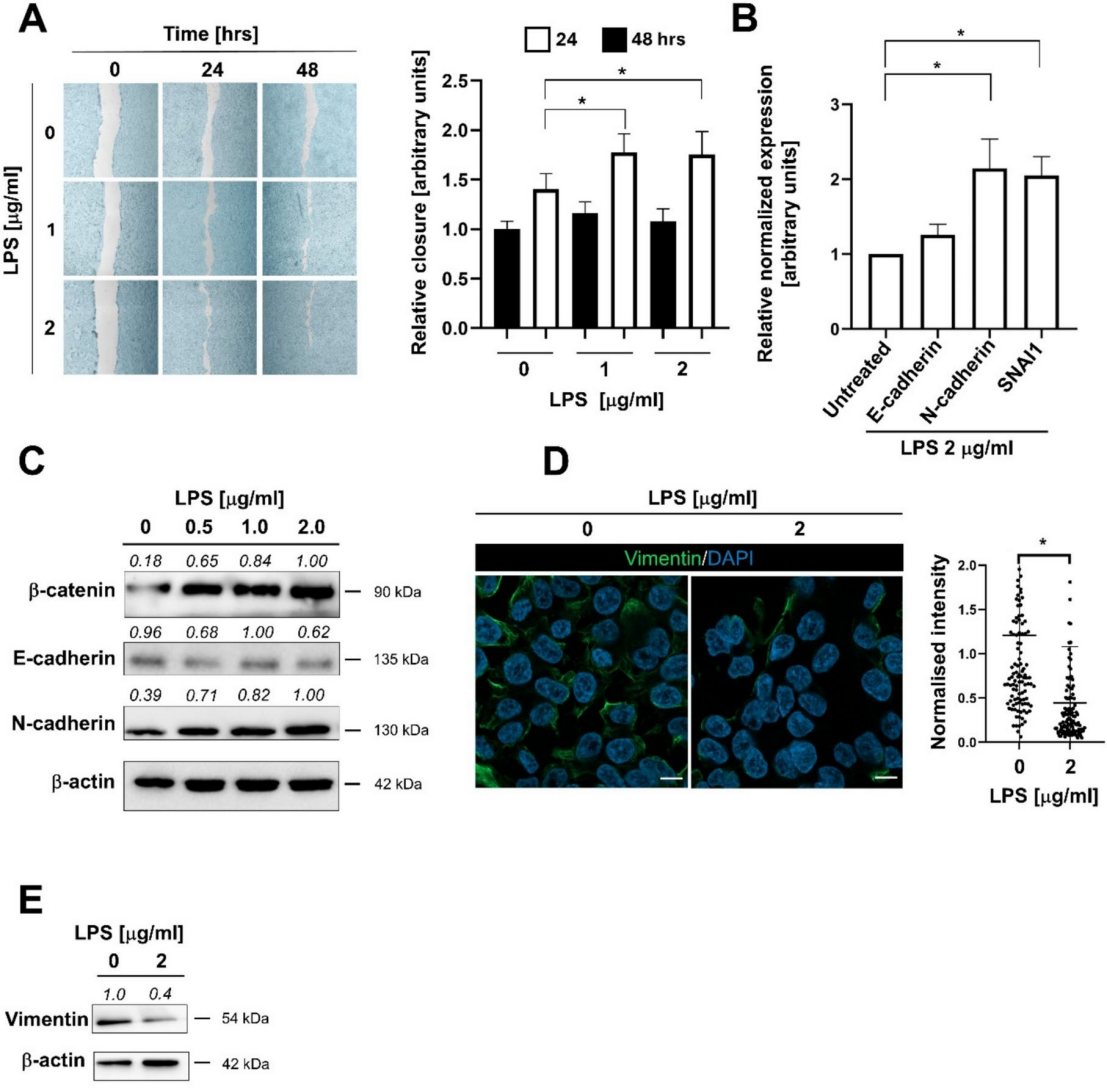
**A** LPS significantly enhances ELEP migration. Migration was assessed by measuring the ability of ELEPs to close a gap in a confluent cell monolayer at the indicated time intervals, and the results were quantified. Asterisks denote statistical significance (*p* < 0.05). **B** mRNA expression levels of E-cadherin, N-cadherin, and SNA1. ELEPs were treated with LPS for 24 h, followed by total RNA extraction, reverse transcription, and qRT-PCR analysis using the ΔΔCt method. Data are presented as mean relative expression normalized to GAPDH ± SD from three independent experiments. **C** Intracellular levels of β-catenin, E-cadherin, and N-cadherin in ELEPs exposed to LPS for 24 h, determined by Western blotting. **D** Vimentin expression is downregulated in ELEPs treated with LPS for 24 h, as visualized and quantified by immunofluorescent microscopy. Scale bars indicate 10 µm. **E** Western blot analysis showing vimentin downregulation in ELEPs exposed to LPS for 24 h. Observed molecular weights and calculated band intensities are indicated. Asterisks denote statistical significance (*p* < 0.05)

To investigate alterations of ELEP phenotype in detail, we calculated the proliferation rate and performed a cell migration assay to evaluate the capacity to close a gap in the cell monolayer. While the proliferation rate was not significantly changed in untreated and LPS-treated ELEPs within 24 and 48 h (Fig. S2A), the LPS-treated ELEPs show an increased migration rate (Fig. 1A), suggesting that LPS stimulates functional alterations in epithelial phenotype. The enhanced migration rate prompted us to further investigate the expression of key adhesion molecules associated with epithelial and mesenchymal phenotypes and EMT. Quantitative RT-PCR and western blotting revealed that the intracellular levels of N-cadherin and SNA1 were significantly upregulated, while the level of E-cadherin remained unchanged (Fig. 1B, C). In parallel, β-catenin levels were clearly increased following LPS treatment. Interestingly, the mesenchymal marker vimentin was downregulated, as confirmed by immunofluorescence microscopy and western blotting (Fig. 1D, E).

**Fig. 2:**
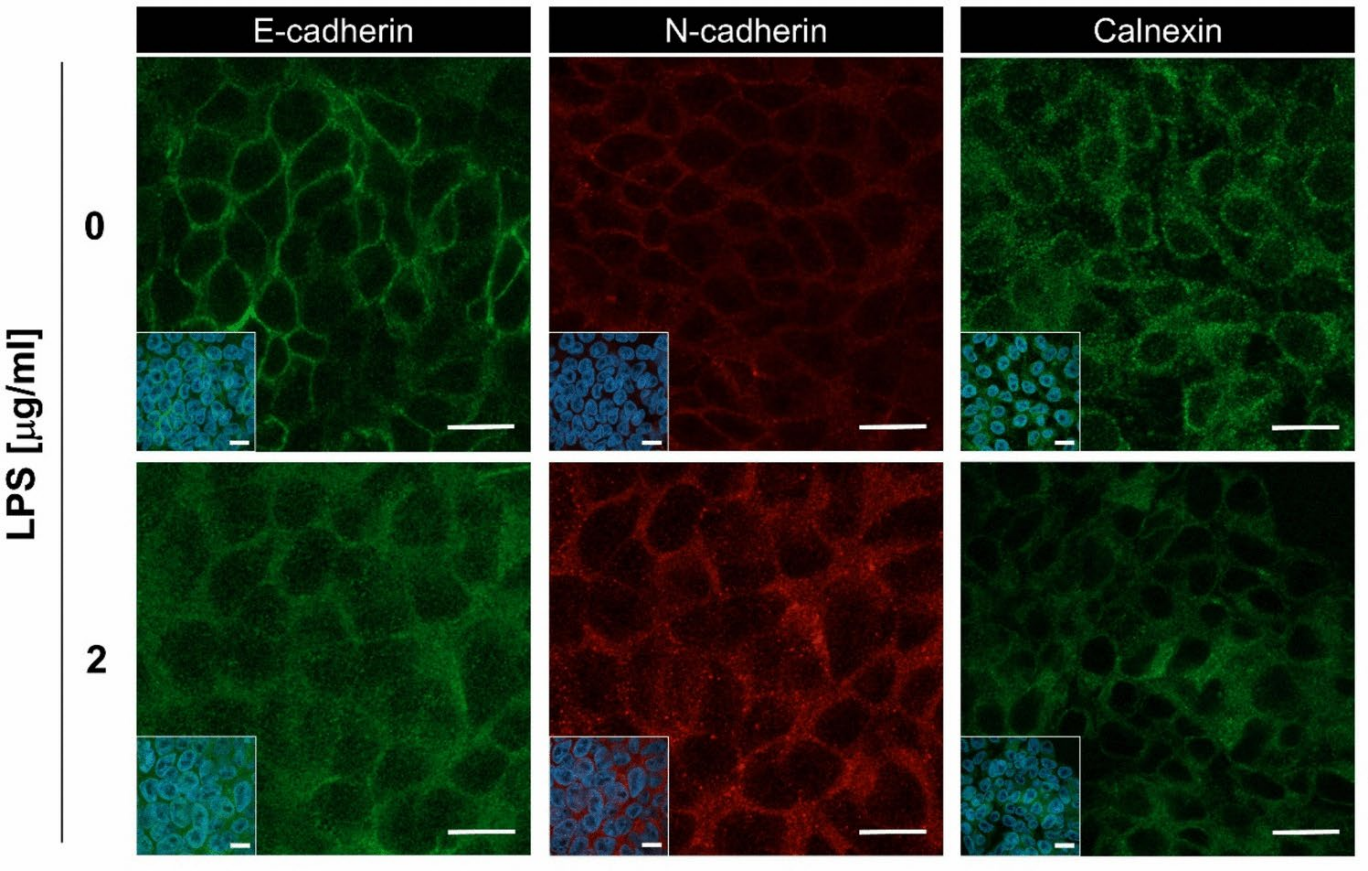
Intracellular levels and distribution of E-cadherin, N-cadherin, and calnexin. ELEPs were cultured for 24 h either in the presence or absence of LPS and analyzed by immunofluorescent microscopy. Insets represent global views of regions showing merged fluorescence from DAPI staining and antibody labeling. The scale bars indicate 10 μm

Then, to examine the expression and subcellular localization of E-cadherin and N-cadherin, we performed immunofluorescent confocal microscopy. While the N-cadherin was clearly upregulated and localized to the cell membrane upon LPS treatment, the signal of E-cadherin in untreated and LPS-treated ELEPs showed similar intensity, but interestingly, different intracellular localization patterns. The untreated ELEPs localized E-cadherin clearly to the cell membrane, but in the LPS-treated ELEPs the E-cadherin was distributed within the cell cytoplasm and cisterns of endoplasmic reticulum (ER) revealed by staining for calnexin, the integral ER protein marker (Fig. 2).

**Fig. 3:**
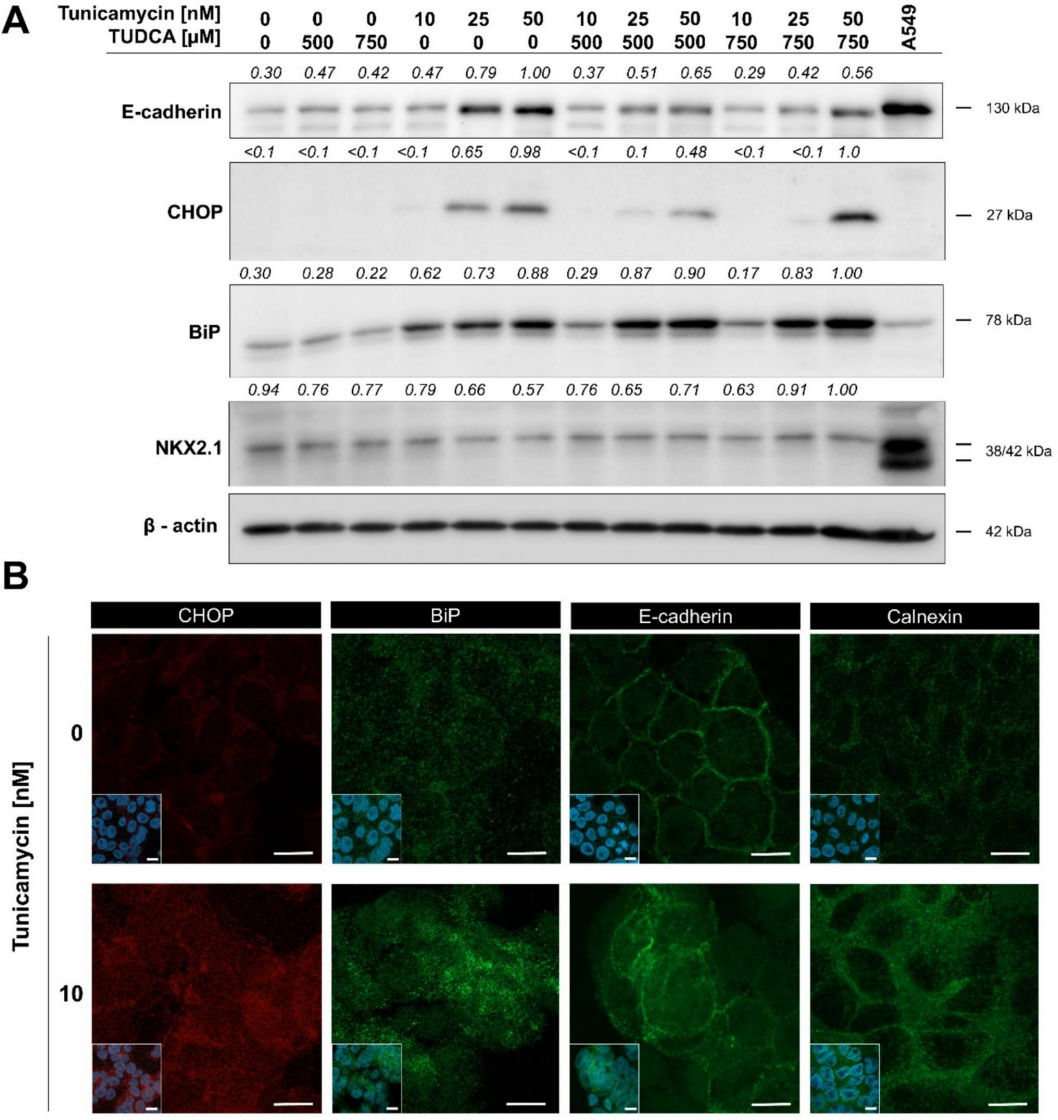
**A** ELEPs upregulate intracellular levels of E-cadherin, CHOP, and BiP while maintaining NKX2-1 expression upon ER stress induction. Cells were treated for 24 h with tunicamycin to induce ER stress and TUDCA to attenuate it, followed by Western blot analysis. Observed molecular weights and relative band intensities are indicated. The A549 lung cancer cell line was included as a positive control.** B** Immunofluorescence microscopy showing intracellular levels and distribution of CHOP, BiP, E-cadherin, and calnexin in ELEPs following 24-h tunicamycin treatment. Insets represent merged DAPI and antibody labeling. Scale bars indicate 10 µm

To determine whether the E-cadherin localization pattern was indeed specific to ER functions, we specifically induced the ER-stress by tunicamycin-dependent inhibition of N-glycosylation and processed the cells for western blotting and immunofluorescent microscopy. We found that ELEPs were highly sensitive to tunicamycin at concentrations as low as 10 nM (Fig. S4) and elevated the ER-stress markers BiP and CHOP (Fig. 3A, B). The expression of NKX2-1 was not significantly altered by tunicamycin treatment. The effect of tunicamycin was partially rescued by attenuation of ER-stress by tauroursodeoxycholic acid (TUDCA). This documented that ELEPs are capable of induction and modulation of the UPR while maintaining the pulmonary identity. Tunicamycin induced upregulation of total E-cadherin protein in dose dependent manner, suggesting accumulation of misfolded protein in the ER (Fig. 3A, S4).

Visualization of E-cadherin and calnexin intracellular localization into ER then revealed a similar distribution pattern in the area corresponding with ER cisterns as in the LPS-treated cells, suggesting that the ER-stress is indeed a candidate molecular mechanism for E-cadherin retention in the ER cisterns (Fig. 3B). Failure of E-cadherin to reach the cytoplasmic membrane can thus explain the decreased intercellular adherence and increased migration upon exposure to LPS.

**Fig. 4:**
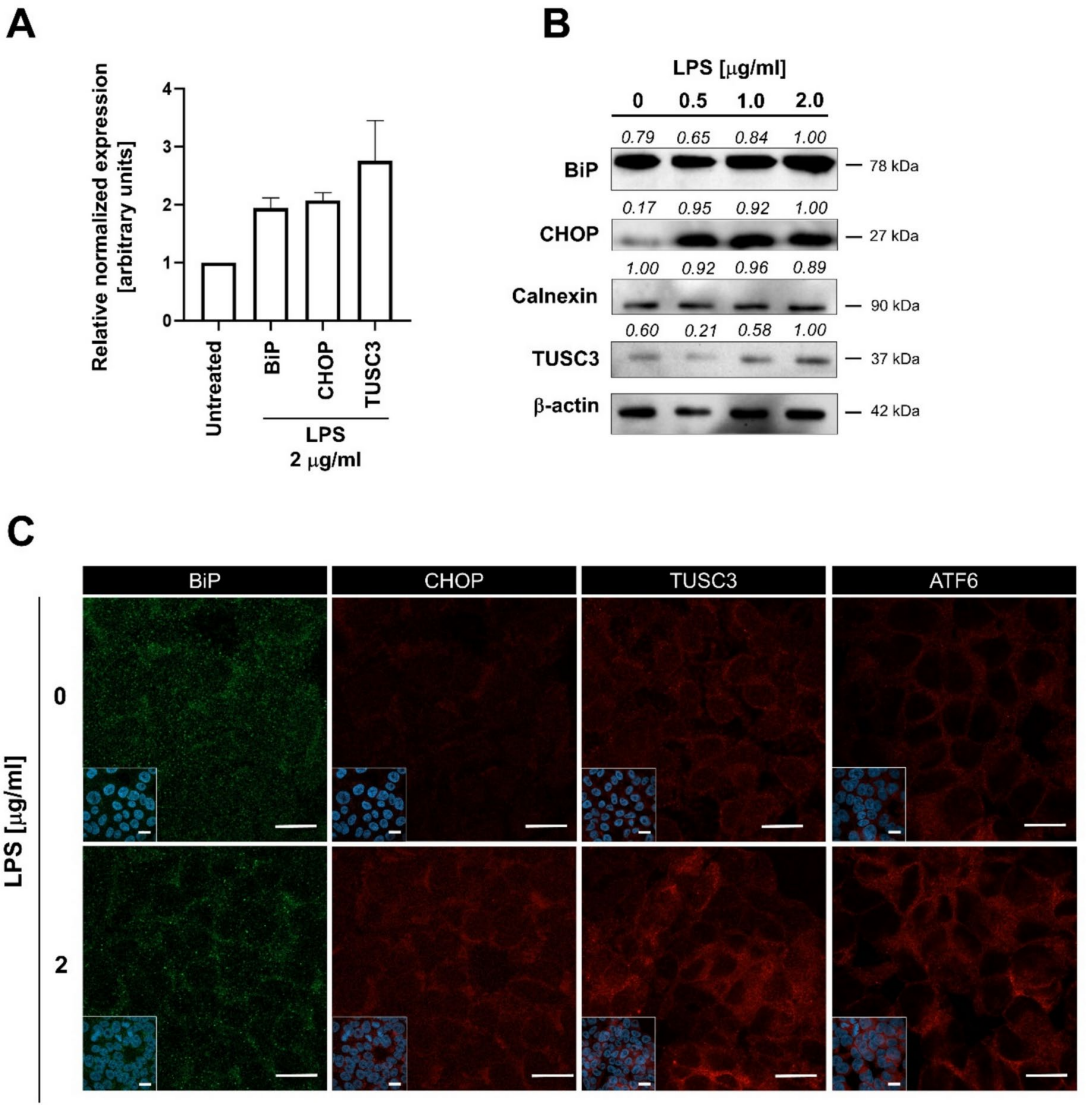
**A** mRNA expression levels of BiP, CHOP, and TUSC3 in ELEPs treated with LPS for 24 h, assessed by qRT-PCR following RNA extraction and reverse transcription. Data represent GAPDH-normalized mean expression ± SD from three independent experiments. **B** Intracellular protein levels of BiP, CHOP, calnexin, and TUSC3 in ELEPs exposed to increasing concentrations of LPS for 24 h, determined by Western blot analysis. **C** Immunofluorescence microscopy showing intracellular levels and subcellular distribution of BiP, CHOP, TUSC3, and ATF6 in ELEPs following 24-h LPS treatment. Insets represent merged images of DAPI staining and antibody labeling. Scale bars indicate 10 µm

Therefore, we investigated whether the LPS-induced retention of E-cadherin was linked to the functional state of the ER. To determine intracellular levels of both canonical UPR (BiP, CHOP, ATF6) and non-canonical markers (TUSC3) we performed the qRT-PCR, western blotting, and immunofluorescent microscopy. We revealed elevated expression of mRNAs coding for BiP, CHOP, and TUSC3 (Fig. 4A) similar to tunicamycin-treated ELEPs, and significantly increased levels of CHOP, TUSC3, and ATF6 proteins upon LPS treatment (Fig. 4B, C). The expression patterns of E-cadherin and N-cadherin in LPS- and tunicamycin-treated ELEPs determined by qRT-PCR correlated with upregulation of total protein levels (Fig. S5).

**Fig. 5:**
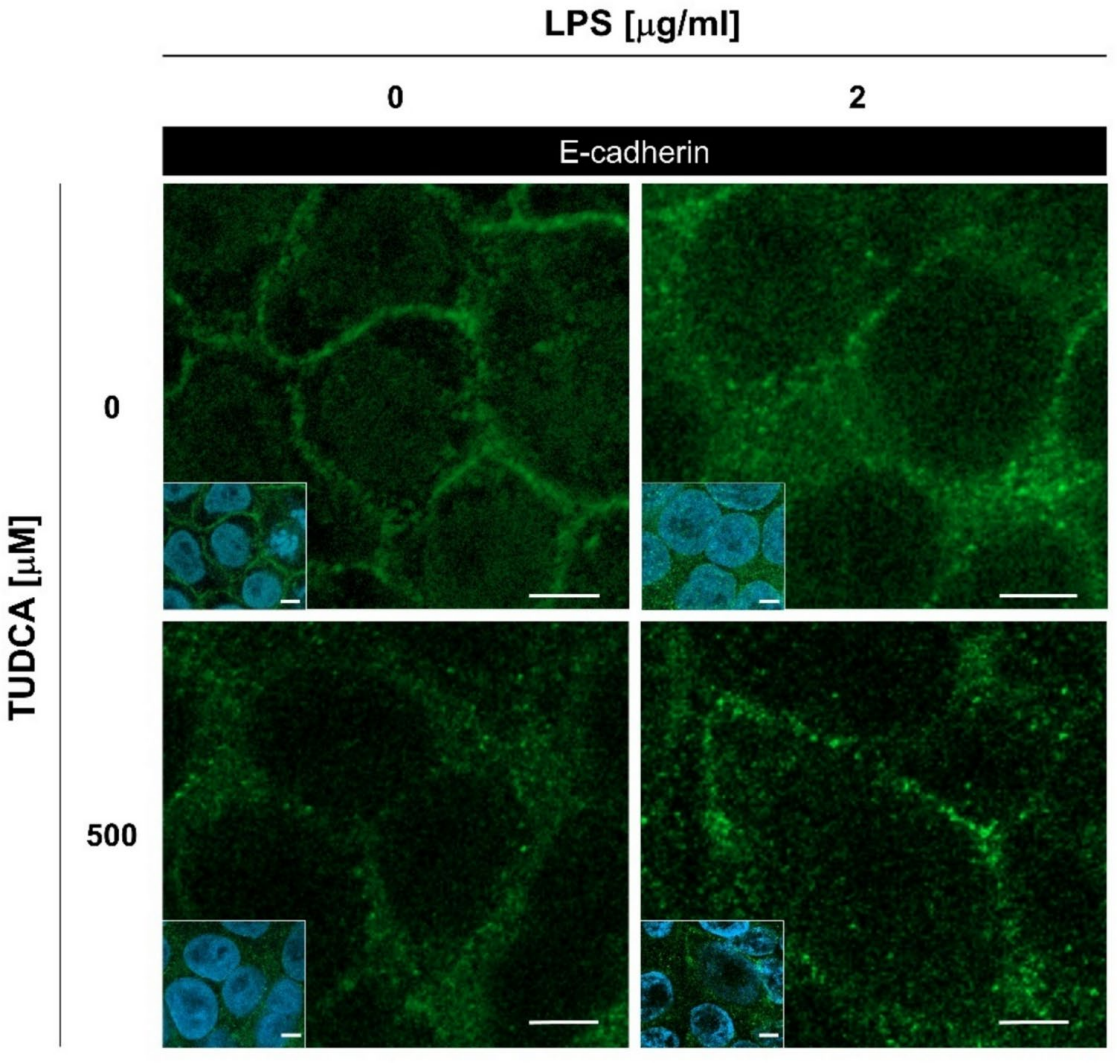
Intracellular localization of E-cadherin in ELEPs treated with LPS, TUDCA, or their combination for 24 h, visualized by immunofluorescence microscopy. Insets represent merged images of DAPI nuclear staining and E-cadherin immunolabeling. Scale bars indicate 10 µm

Attenuation of ER stress by the well-established ER-stress inhibitor, tauroursodeoxycholic acid (TUDCA), led to a decrease in the transcription rates of the BiP, CHOP, and TUSC3 genes. (Fig. S4). Importantly, co-treatment with TUDCA reversed the retention of E-cadherin in the ER and partially rescued its localization to the cytoplasmic membrane (Fig. 5).

**Fig. 6:**
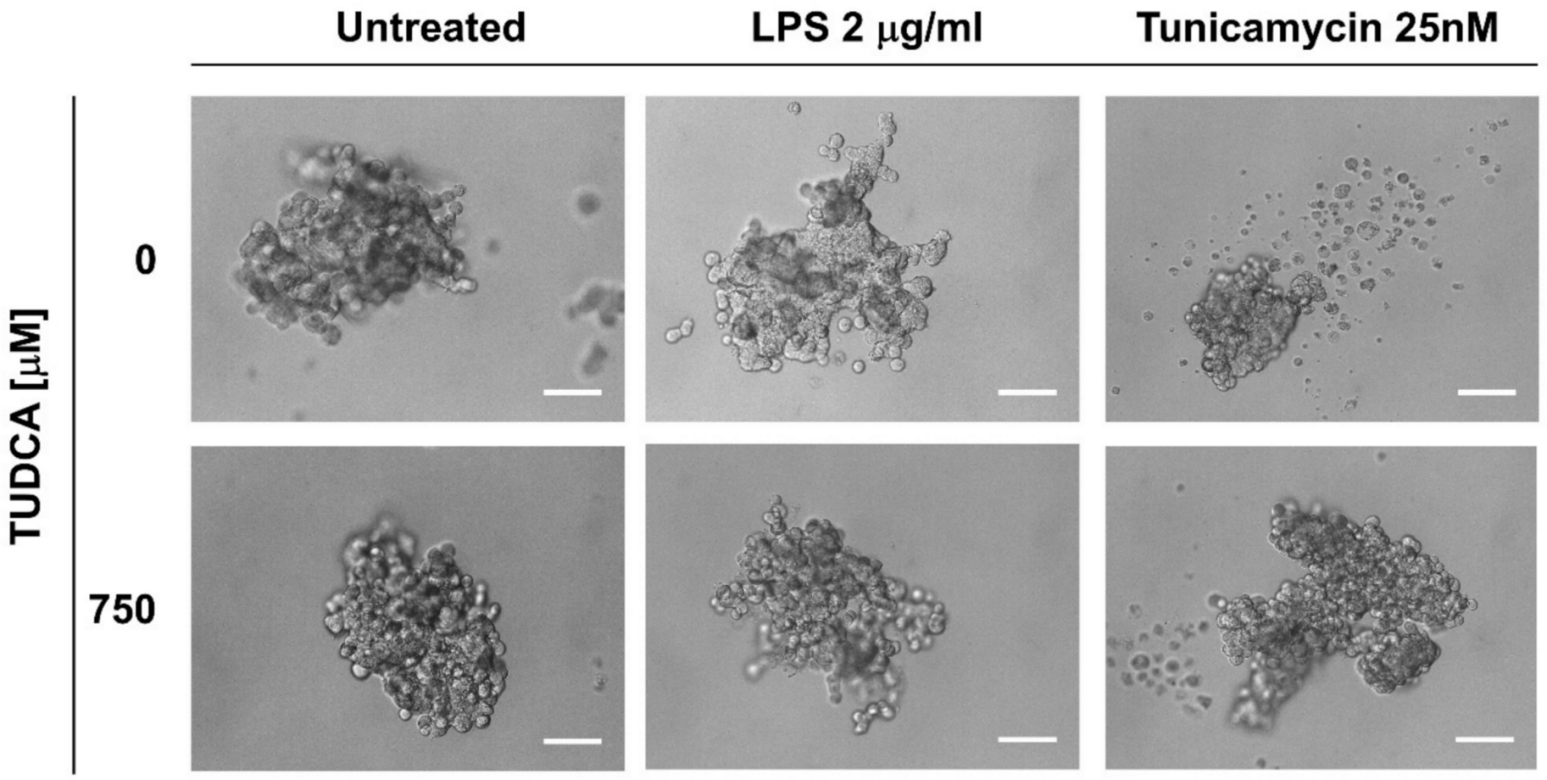
Representative images of ELEP-derived spheroids exposed to LPS (2 µg/mL) or tunicamycin (25 nM), with or without co-treatment with TUDCA (750 µM). Scale bars indicate 200 µm

To validate the effects of TUDCA on alterations to the ELEP phenotype induced by LPS, we established a series of ELEP spheroids and exposed them to LPS or tunicamycin, with or without TUDCA for 48 h. While the untreated ELEPs maintained a relatively compact spheroid morphology, LPS-treated spheroids were significantly more irregular, with partially branched protrusions. Tunicamycin-treated spheroids showed reduced growth, disintegrated, and produced large amounts of loose cells and cell debris. Interestingly, co-treatment with TUDCA partially reversed the effects induced by LPS and tunicamycin, resulting in more compact spheroids (Fig. 6).

**Fig. 7:**
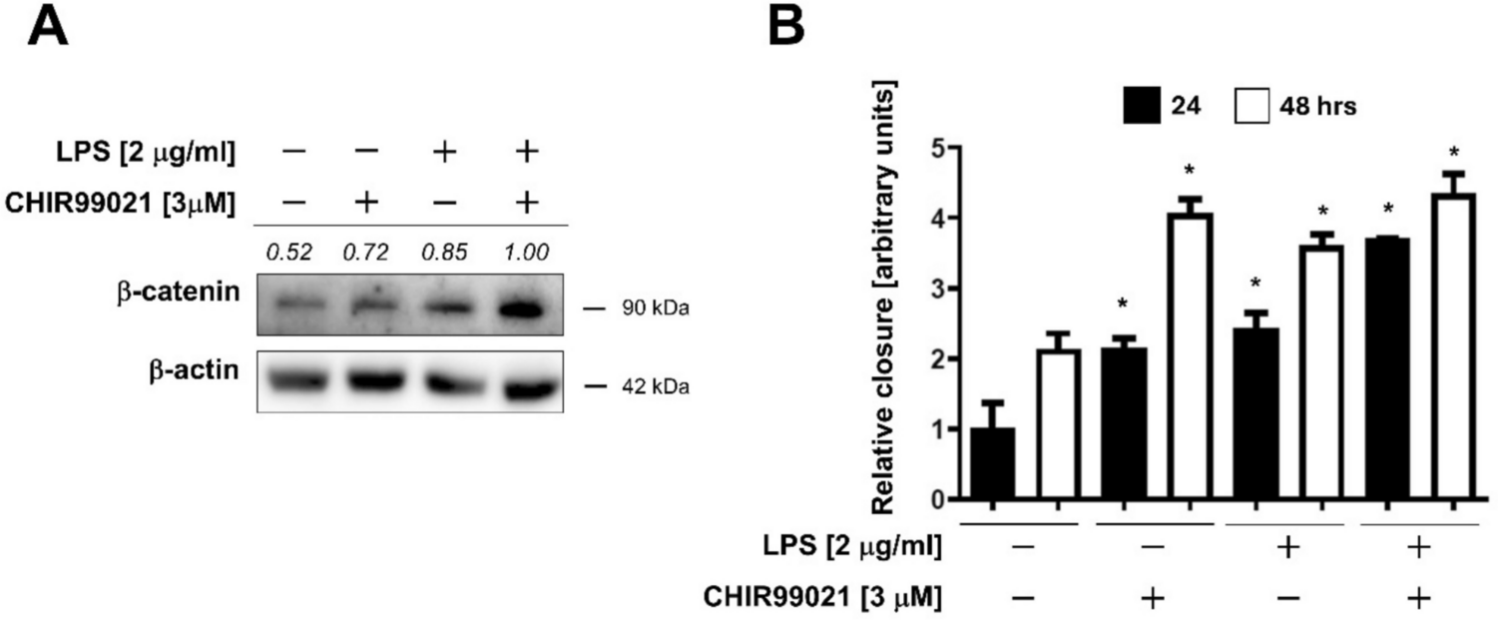
**A** Intracellular β-catenin levels in ELEPs treated with LPS (2 µg/mL), CHIR99021 (3 µM), or their combination for 24 h, as determined by Western blot analysis. Observed molecular weights and corresponding band intensities are indicated. **B** Quantification of ELEP cell migration following treatment with LPS (2 µg/mL), CHIR99021 (3 µM), or their combination for 24 and 48 h. Asterisks denote statistically significant differences (*p* < 0.05) between 24 and 48 h control

E-cadherin binds β-catenin and interacts with actin cytoskeleton to maintain cell adhesion. Consistent with our results, β-catenin levels were increased following LPS treatment (Fig. 1C). To determine whether β-catenin was involved in the enhanced migration and reduced adhesion in our ELEP cells, we inhibited the glycogen synthase kinase-3 (GSK3) by CHIR99021 and determined β-catenin levels by western blotting. As expected, the inhibition of GSK3 stabilized intracellular levels of β-catenin (Fig. 7A). Then we analyzed the ELEPs for migration and revealed that CHIR99021 clearly enhanced the closure of scratched area in a similar extent to LPS and augmented the ELEP migration stimulated by LPS (Fig. 7B and Fig. S6). In summary, ELEPs respond to LPS exposure by induction of UPR, retention of E-cadherin in ER, and enhanced migration through β-catenin upregulation.

## Discussion

Bacterial endotoxins, such as LPS, are major drivers of lung inflammation. The effects of LPS on the immune [25, 26] and endothelial cells [27] are well known, involving the production of a range of immunomodulatory molecules and the development of a pro-inflammatory microenvironment. Direct effects of LPS on pulmonary tissues are executed through the TLR4 receptor [28] and downstream MyD88- and TRIF-dependent pathways, and production of pro-inflammatory cytokines (e.g., IL-6, TNF-α) and chemokines (e.g., IL-8, CCL2). LPS then directly or through the formation of the pro-inflammatory microenvironment stimulates the stromal fibroblasts to produce extracellular matrix (ECM) [29] and reactive oxygen species [30], promotes endothelial activation and cell death [31], and increases the propensity to malignant transformation of lung epithelia [32]. LPS was also reported to stimulate the immune escape of lung cancer cells [33] or contribute to metastatic dissemination [34, 35]. In alveolar epithelia, Wu recently described the induction of EMT by LPS in the A549 lung adenocarcinoma cell line [36]. Similarly, Ding et al. reported the involvement of the PI3K/GSK-3β signaling pathway in LPS induced morphological changes of the A549 cell line [30]. In the LPS-induced in vivo acute lung injury model, the lung tissues upregulated components of canonical UPR and attenuation of ER-stress increased cell survival in airway epithelia [37]. In other epithelia, such as intestinal or in salivary glands, LPS was described to increase the permeability of tight junctions [38] or deregulate the electrolyte gradients, leading to loss of barrier functions [39]. LPS isolated from E. coli was shown to induce phenotypic shifts and EMT in A549 cancer cell lines or primary mouse lung tissue, affecting expression of E cadherin and BAMCI. Interestingly, LPS of different bacterial origin (e.g. *Bacteroides vulgatus*) prevented E. coli LPS-induced transition, suggesting complex receptor interactions and molecular response [40].

Endoplasmic reticulum has been traditionally associated with synthesis, initial post-translational modification, and quality control of membrane and secreted proteins [41]. In addition, ER represents a critical signaling hub integrating intrinsic and extrinsic stimuli into a global molecular or cellular response in organ development or tissue regeneration [17]. Using various cancer and non-cancer models, we described previously that epithelial cells surviving the ER-stress state alter significantly their phenotype [42, 43].

Here we envisage that attenuation of E-cadherin trafficking through ER and Golgi apparatus induced by LPS can represent a novel mechanism of LPS-stimulated shifts of epithelial phenotypes in septic or injured lungs. However, the vast majority of reports come from established cancer cell lines or heterogeneous primary lung tissue. To address the effects of LPS in pure, non-cancerous cell populations, we used the newly established, pluripotent stem cells-derived-ELEP cells that possess molecular and structural hallmarks of immature alveolar type II pneumocytes and maintain a high proliferation rate in vitro. Interestingly, while the ELEPs show a clear epithelial morphology and molecular pattern of alveolar pneumocytes, such as NKX2-1, they also express high basal rates of N-cadherin and vimentin. LPS further upregulates intracellular levels of N-cadherin, but in parallel downregulates the vimentin, suggesting that ELEP are prone to hybrid EMT with a propensity to acquire migratory phenotype if properly stimulated. Importantly, shifts in ELEP phenotype triggered by UPR can be attenuated by chemical chaperones like TUDCA. The hybrid EMT is an emerging phenomenon in cancer that can explain the substantial phenotypic heterogeneity, first described in metastasizing epithelial tumors (for the state-of-the-art review see [44]). In lung cancer, the hybrid-EMT cells interact differently with NK cells and alter the immunosurveillance through specific secreted chemokine patterns [16]. Recently, Lobb et al. reported that EMT-induced cell depolarization and loss of adhesion can also affect protein composition within secreted small extracellular vesicles, providing a clinically significant predictive pattern in non-small cell lung cancer patients [45]. The interaction between E-cadherin and β-catenin is crucial for maintaining epithelial integrity. Interestingly, LPS treatment led to increased β-catenin levels, which may contribute to the observed migratory phenotype. Stabilization of β-catenin through GSK3 inhibition further enhanced migration, indicating its functional role in this process. While β-catenin is primarily known for its role in Wnt signaling and transcriptional regulation, it can also promote migration independently of transcription by modulating protein interactions and degradation of adhesion molecules in cancer cells (for review see [46]).

In summary, our findings reveal that exposure to purified *E. coli* LPS promotes the acquisition of a hybrid EMT phenotype in ELEPs through the induction of ER stress, retention of E-cadherin in the ER, and upregulation of β-catenin. Targeting pathways regulating protein synthesis and trafficking, such as UPR can therefore provide an alternative mechanism for an explanation of LPS-induced remodeling of lung tissue, as well as a pharmacologically attractive molecular target for various lung pathologies.

## Supplementary Information

Below is the link to the electronic supplementary material.

Supplementary file1 (DOCX 17 kb)

Supplementary file2 (DOCX 1507 kb)

## Data Availability

Data is provided within the manuscript or supplementary information files.
