# Supplementary material for: Lipopolysaccharide induces retention of E-cadherin in the endoplasmic reticulum and promotes hybrid epithelial-to-mesenchymal transition of human embryonic stem cells-derived expandable lung epithelial cells
Source: Inflamm Res. 2025 May 24;74(1):82. doi: 10.1007/s00011-025-02041-4 (PMC12103375; doi:10.1007/s00011-025-02041-4)
Supplement: Supplementary file 1 — Supplementary file1 (DOCX 17 kb) [file 11_2025_2041_MOESM1_ESM.docx]

| **Gene** | **Forward Primer** | **Reverse Primer** | **Tm (°C)** | **Primer length (nt)** | **Amplicon Size (bp)** |
| --- | --- | --- | --- | --- | --- |
| **SNAI1** | **5'-TCGGAAGCCTAACTACAGCGA-3'** | **5'-AGATGAGCATTGGCAGCGAG-3'** | **60.9 / 60.8** | **21 / 20** | **140** |
| **BiP** | **5'-AGCTGTAGCGTATGGTGCTG-3'** | **5'-AAGGGGACATACATGAAGCAGT-3'** | **59 / 59** | **20 / 22** | **91** |
| **CHOP** | **5'-GGAGCATCAGTCCCCCACTT-3'** | **5'-TGTGGGATTGAGGGTCACATC-3'** | **60 / 60** | **20 / 21** | **101** |
| **E-cadherin** | **5'-TGGAGGAATTCTTGCTTTGC-3'** | **5'-CGCTCTCCTCCGAAGAAAC-3'** | **60 / 60** | **20 / 19** | **64** |
| **N-cadherin** | **5'-GGTGGAGGAGAAGAAGACCAG-3'** | **5'-GGCATCAGGCTCCACAGT-3'** | **60 / 59** | **21 / 18** | **72** |
| **TUSC3** | **5'-CCCACACAATGGACAAGTGA-3'** | **5'-TGTGTGATTCTGCCACAAACT-3'** | **60 / 59** | **20 / 22** | **68** |
| **GAPDH** | **5'-TCTGCTCCTCCTGTTCGACA-3'** | **5'-CCCAATACGACCAAATCCGT-3'** | **59.9 / 60** | **20 / 20** | **117** |

**Lipopolysaccharide induces retention of E-cadherin in the endoplasmic reticulum and promotes hybrid epithelial-to-mesenchymal transition of human embryonic stem cells-derived expandable lung epithelial cells.**

Türkan Portakal^1^, Vítězslav Havlíček^1^, Jarmila Herůdková^1,3^, Vendula Pelková^1,3^, Riza Can Cakmakci^1^, Tereza Gruntová^1^, Aleš Hampl^1,2,3^, Petr Vaňhara^1,2,3*^

^1^Department of Histology and Embryology, Faculty of Medicine, Masaryk University, Kamenice 753/5, 625 00 Brno, Czech Republic

^2^International Clinical Research Center, St. Anne’s University Hospital, Brno, Czech Republic

^3^University Hospital Brno, Jihlavská 340/20, Brno, Czech Republic

*author for correspondence: Petr Vaňhara, [pvanhara@med.muni.cz](mailto:pvanhara@med.muni.cz), tel.: +420 54949 7780

**Supplementary Table 1.**

Sequences and principal parameters of qRT-PCR primers.
