# Supplementary material for: Lipopolysaccharide induces retention of E-cadherin in the endoplasmic reticulum and promotes hybrid epithelial-to-mesenchymal transition of human embryonic stem cells-derived expandable lung epithelial cells
Source: Inflamm Res. 2025 May 24;74(1):82. doi: 10.1007/s00011-025-02041-4 (PMC12103375; doi:10.1007/s00011-025-02041-4)
Supplement: Supplementary file 2 — Supplementary file2 (DOCX 1507 kb) [file 11_2025_2041_MOESM2_ESM.docx]

**Lipopolysaccharide induces retention of E-cadherin in the endoplasmic reticulum and promotes hybrid epithelial-to-mesenchymal transition of human embryonic stem cells-derived expandable lung epithelial cells**

Türkan Portakal^1^, Vítězslav Havlíček^1^, Jarmila Herůdková^1,3^, Vendula Pelková^1,3^, Tereza Gruntová^1^, Riza Can Cakmakci^1^, Hana Kotasová^1^, Aleš Hampl^1,2,3^, Petr Vaňhara^1,2,3*^

^1^Department of Histology and Embryology, Faculty of Medicine, Masaryk University, Kamenice 753/5, 625 00 Brno, Czech Republic

^2^International Clinical Research Center, St. Anne’s University Hospital, Pekařská 664/53, 602 00, Brno, Czech Republic

^3^University Hospital Brno, Jihlavská 340/20, 625 00, Brno, Czech Republic

*author for correspondence: Petr Vaňhara, [pvanhara@med.muni.cz](mailto:pvanhara@med.muni.cz), tel.: +420 54949 7780

**Supplementary figures**


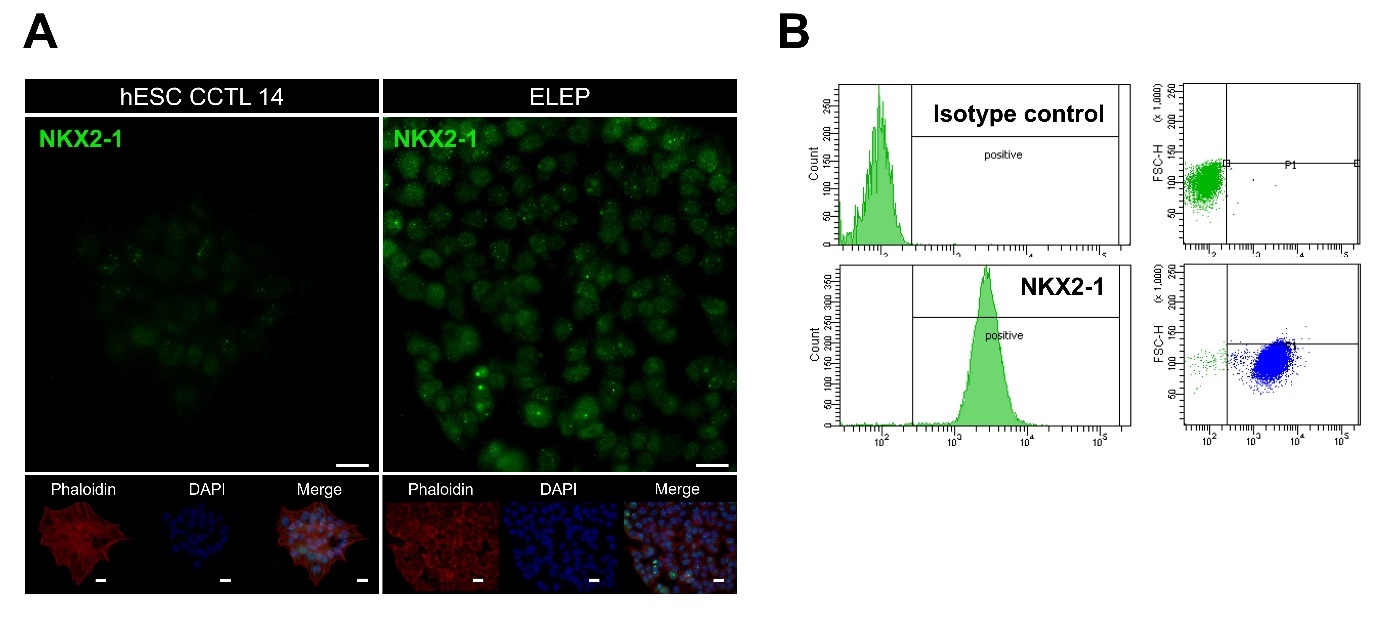


***Fig. S1*** *(****A****)* *ELEPs upregulate the lung-specific transcription factor NKX2-1, as demonstrated by immunofluorescence microscopy and (****B****) flow cytometry. Scale bars indicate 10μm.*


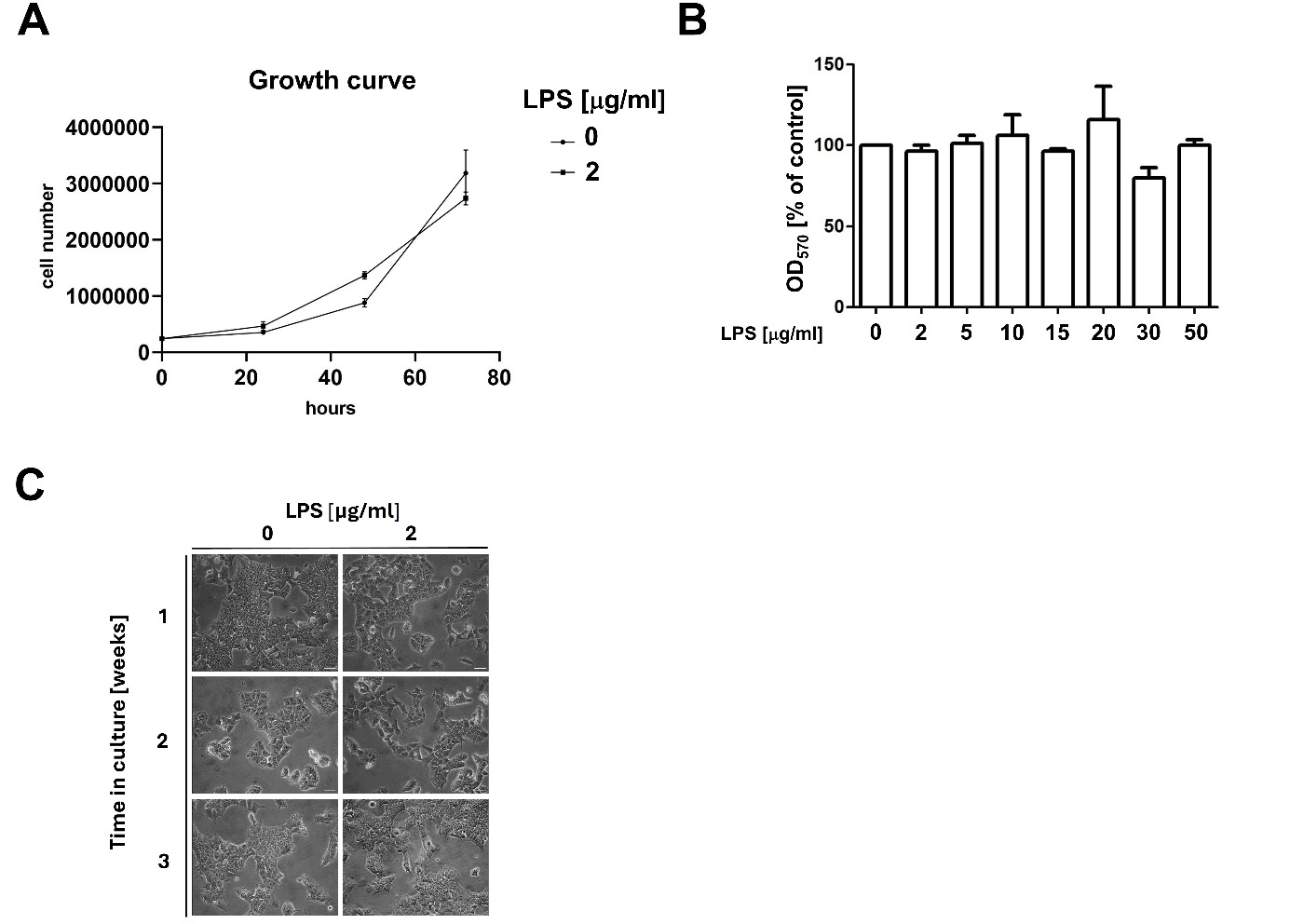


***Fig. S2*** *(****A****) Proliferation rate of ELEPs cultured in the presence or absence of LPS. The plot shows the mean ± SD of three independent experiments. (****B****) LPS does not induce ELEP cell death across a range of LPS concentrations. ELEPs were cultured in the presence or absence of LPS for 48 hours and cell viability was assessed using the MTT assay. (****C****) Illustrative image documenting negligible effects on cell death in ELEPs exposed to LPS in long-term culture.*


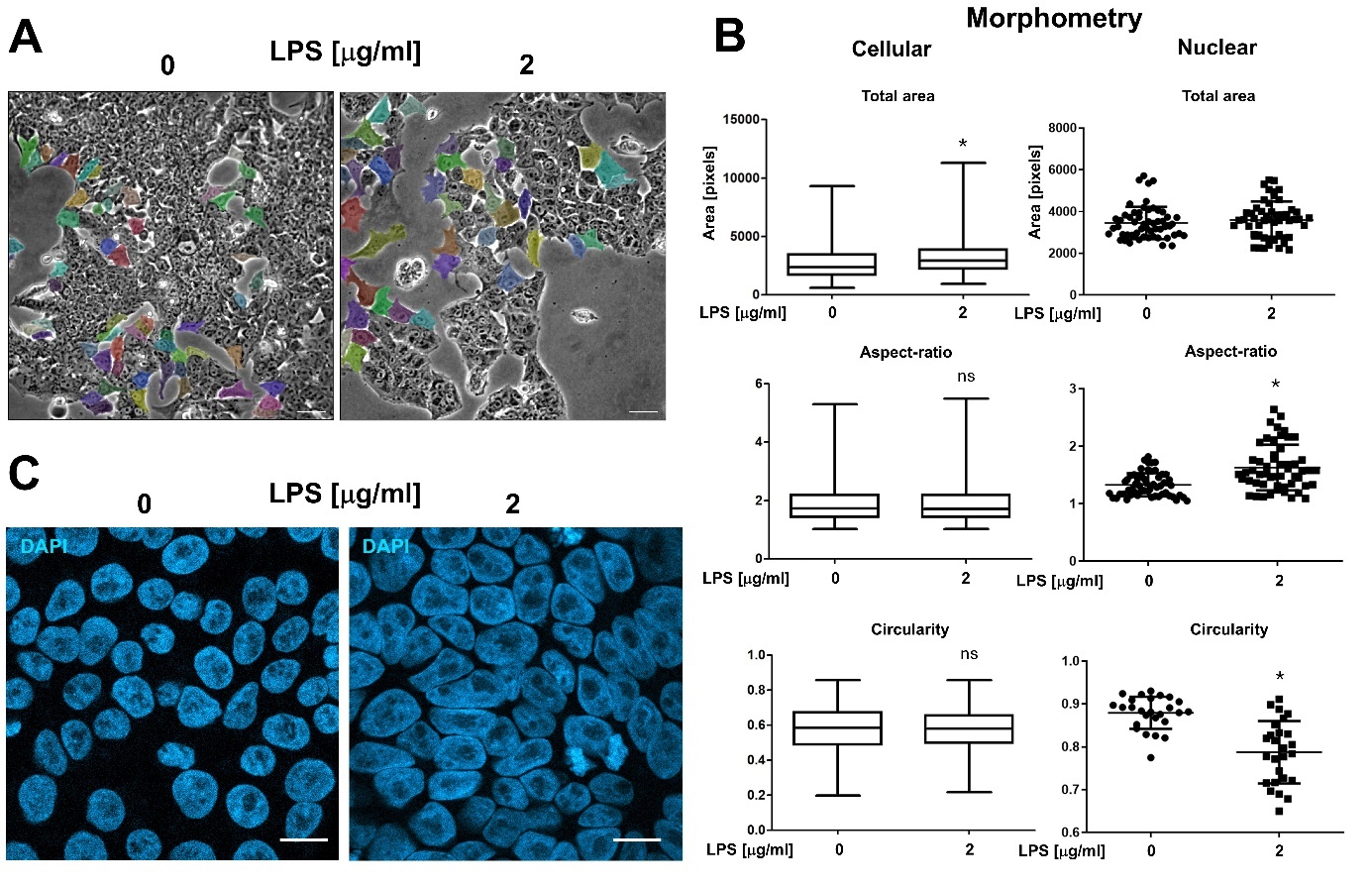


***Fig. S3*** *(****A)*** *LPS decreases the coherence of the ELEP monolayer. ELEPs were cultured for 48 hours in the presence or absence of LPS, and light microscopy images of subconfluent monolayer were analyzed for cell morphometry. Examples of cells included in the analysis are highlighted in color. Scale bars indicate 50 μm. (****B****) Visualization of cellular and nuclear morphometry. Morphometric parameters were quantified using more than 100 and 500 cells for cellular and nuclear analysis, respectively. Asterisks indicate statistical significance at p<0.05. (****C****) Illustrative fluorescent microscopy images of DAPI-stained ELEP nuclei reveal alterations in aspect ratio and circularity. Scale bars indicate 10 μm.*


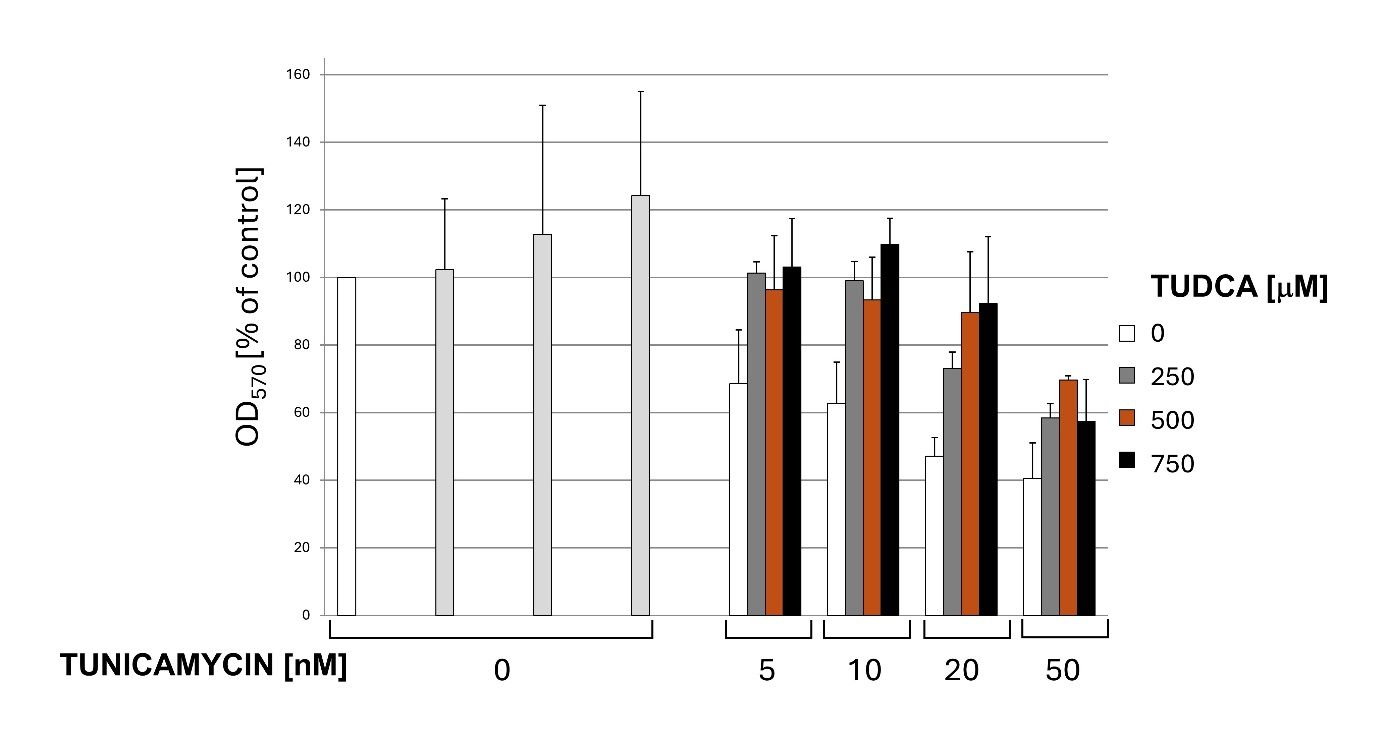


***Fig. S4****. ELEPs show pronounced sensitivity to tunicamycin-induced cell death, which is partially mitigated by TUDCA treatment. ELEPs were treated with tunicamycin and/or TUDCA at the indicated concentrations for 24 hours, and cell viability was determined by the MTT assay.*


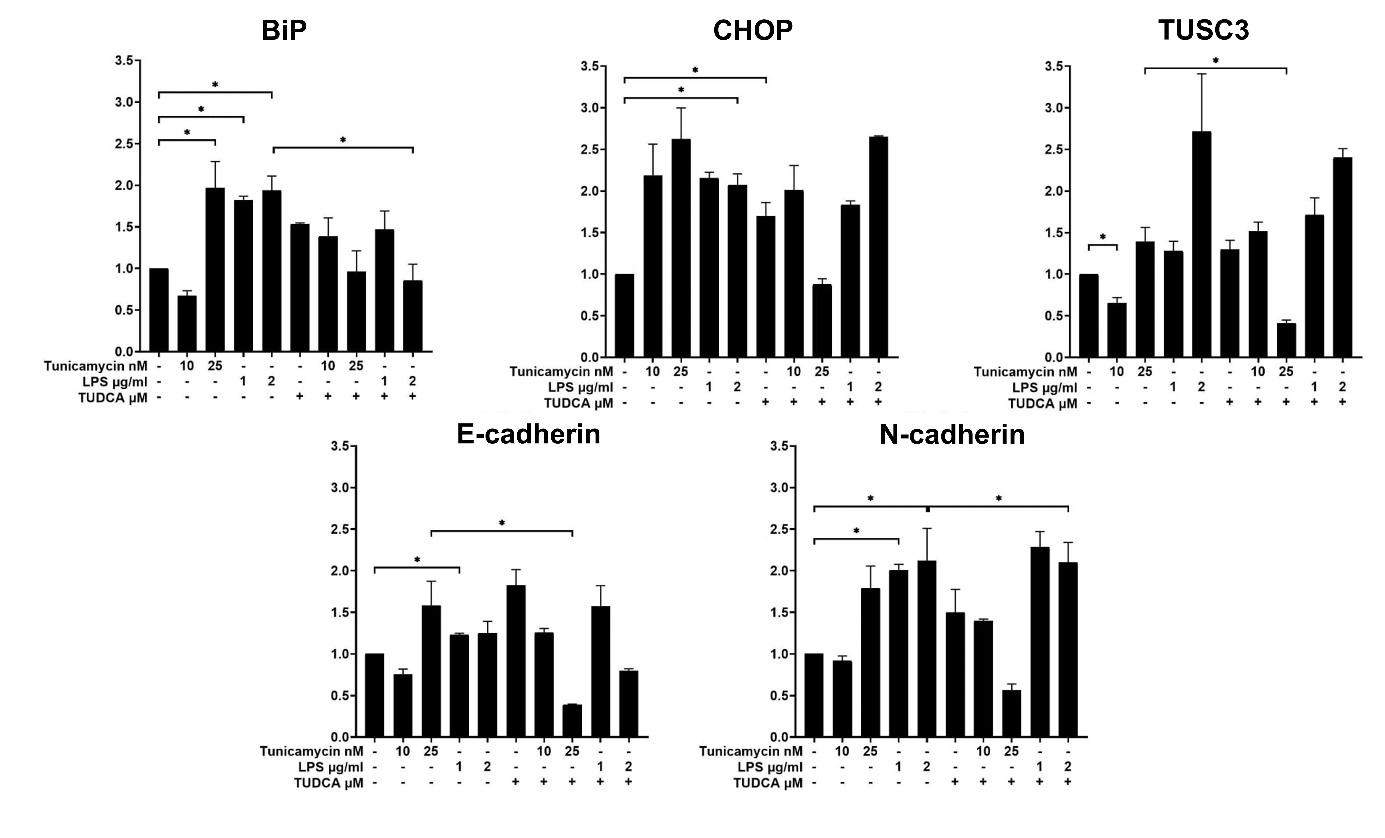


***Fig. S5.*** *Expression of BiP, CHOP, TUSC3, E-cadherin, and N-cadherin mRNA in ELEPs treated with LPS for 24 hours. Total RNA was extracted, reverse transcribed, and analyzed by qRT-PCR. Data are presented as mean relative expression normalized to GAPDH ± SD from three independent experiments.*

*
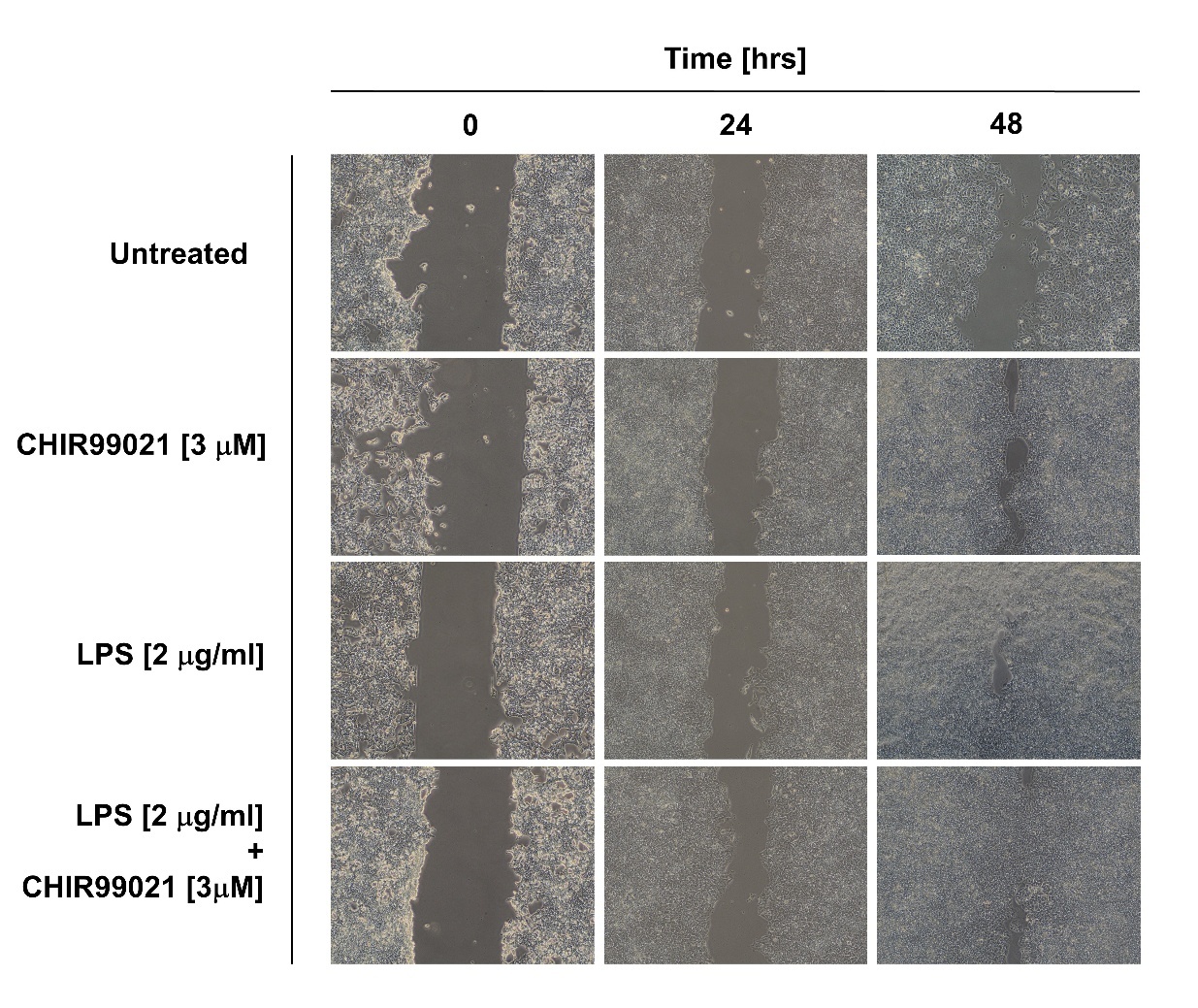
*

***Fig. S6*** *Representative image showing ELEP cell migration following treatment with LPS (2 µg/mL), CHIR99021 (3 µM), or their combination for 24 or 48 hours.*
